# Context-Based Tourism Information Filtering with a Semantic Rule Engine

**DOI:** 10.3390/s120505273

**Published:** 2012-04-26

**Authors:** Carlos Lamsfus, David Martin, Aurkene Alzua-Sorzabal, Diego López-de-Ipiña, Emilio Torres-Manzanera

**Affiliations:** 1 Competence Research Center in Tourism, CICtourGUNE, Mikeletegi Pasealekua 56, Donostia - San Sebastián 94348, Spain; E-Mail: davidmartin@tourgune.org; 2 Faculty of Engineering, University of Deusto, Avda. de las Universidades 24, Bilbao 48007, Spain; E-Mails: aurkenealzua@tourgune.org (A.A.S.); dipina@deusto.es (D.L.-I.); 3 Department of Statistics, University of Oviedo, Avda. Luis Moya 261, Gijón 33003, Spain; E-Mail: torres@uniovi.es

**Keywords:** context-awareness, push technology, tourism, reasoning, semantic-web, digital broadcasting

## Abstract

This paper presents the CONCERT framework, a push/filter information consumption paradigm, based on a rule-based semantic contextual information system for tourism. CONCERT suggests a specific insight of the notion of context from a human mobility perspective. It focuses on the particular characteristics and requirements of travellers and addresses the drawbacks found in other approaches. Additionally, CONCERT suggests the use of digital broadcasting as push communication technology, whereby tourism information is disseminated to mobile devices. This information is then automatically filtered by a network of ontologies and offered to tourists on the screen. The results obtained in the experiments carried out show evidence that the information disseminated through digital broadcasting can be manipulated by the network of ontologies, providing contextualized information that produces user satisfaction.

## 1. Introduction

eTourism [1] is becoming an increasingly significant research discipline within Information and Communication Technologies (ICT), especially within ubiquitous computing. Every traveller has his own preferences, requirements and expectations. One of the greatest dangers is to fall back on the traditional way of understanding travellers in a fragmented manner. New trends show that people in general and travellers in particular do not identify with predetermined groups [2]. The challenge is to act accordingly, giving rise to inherent personalisation. Thus, context-based services do not only represent the opportunity and future in the travel and tourism industries [3], but also the possibility to better understand human behaviour in the future digital society.

Current state-of-the-art technology enables contextual computing services in tourism [4–6]. Typically, a tourist is a person in a visiting situation [7], who may be more or less familiar and aware of useful information that is available about the destination. This kind of unfamiliar place and lack of knowledge vary from place to place and from individual to individual. Therefore, these form an important part of the personal context of a visitor in a particular place, who would require some kind of assistance when performing information searches.

However, context has traditionally been studied in other research disciplines [8–10]. Nonetheless, there are various examples of tourism mobile guides [3], in which the notion of context has been directly imported from the previously mentioned fields without specifically addressing the characteristic requirements of context within the domain of travellers. This lack of sufficient theoretic support has lead to an oversimplified understanding of context. Thus, understanding the notion of context within the framework of human mobility is essential not only to derive a sound model for computing devices to process context information in a meaningful way [11], but also to delimit and elucidate the notion of context in tourism.

In addition, traditional context-aware applications rely on networks of sensors to collect contextual information, which makes them dependent on the particular infrastructure needed to gather contextual information, their hardware infrastructure and corresponding communication protocols [12]. Most of these are pull-based applications and thus require intensive human intervention. In the case of tourism this manipulation is somewhat restricted due to the small size of screens and keyboards in mobile devices, as well as knowledge about the infrastructure deployed in the new places visited. These appear to be limiting factors when applied to the domain of tourism, where push-based information systems have shown to encourage tourists to use technology-based information systems [4,13].

Motivated by all of the above, this paper presents a specific approach to context-aware information dissemination for travellers based upon a push/filter paradigm, named CONCERT. This framework is built following the statistical recommendations on visitor classification established by the UNWTO [7]. It must fulfil two requisites. The first one is that a network of ontologies approach can be used to model the context of a visitor and provide her with useful information using description logics and rule based reasoning. The second one is that visitors are satisfied with the results. The model is a semantic-based visitor-centred model for context on the realm of tourism. First a theoretical approach to context in the realm of tourism is established. The research work continues by focusing on how a semantic-based rule filtering engine provides visitors with relevant information according to their context. To do so, the Internet has been used as primary source of information for both context and tourism information, and also with digital broadcasting as (*push*) communication technology to disseminate context and tourism information.

The remaining of the paper is structured as follows. Section 2 presents briefly the related work. Section 3 shows the main theoretic research work carried out, as well as its corresponding semantic model. Section 4 describes the system architecture including a thorough explanation of the reasoning process. Section 5 shows the evaluation and finally, Section 6 concludes the paper with some remarks and suggests future research lines.

## 2. Related Work

Context-aware research falls into two categories with the focus on context theory and establishing and developing standard context-aware models and methodologies. Semantic technologies, more precisely ontologies, bridge between these two categories. From a context theory perspective, the work carried out by researchers was basically focused on defining the theoretical and conceptual foundations of context-awareness. They developed a number of applications in order to test their theory, however, the theoretical work carried was so intense that the most relevant definitions of context were put forward throughout those years [14–16].

Analyses of context management methods [10] indicate that ontologies take in adequate functionalities for context information management. Hence, several authors working on context-awareness started to use semantic technologies in order to model context and manage context information. This set the way for systems to share, integrate, exchange and re-use context information, and moreover, it enabled not only checking the models consistency but also inferring implicit context knowledge [17,18]. Researchers conducting this work were primarily focused on finding standard context management methods rather than in the development of the theory that supported them. This research falls into the second category of context-aware research.

Several mobile tourism guide surveys have already been published [3,19,20]. The first two reviews primarily analyse early generation visitor guides and investigate specific issues, such as support for maps or mobility aspects. However, they often do not provide a comprehensive insight into the kinds of services that are supported, and moreover, in depth technical aspects of how these services are delivered to the end-user are also not described. Mobile tourism guides either provide location-based information services or concentrate on delivering personalized information, *i.e.*, they fail to provide a combination of both as a rudimentary context-aware kind of service [3].

There are just two examples of real context-aware mobile tourism guides [4,5]. Interestingly, although the CAIPS system [4] provides rule-based push information, it fails to provide a general framework to support visitors, since it is more focused on creation of rules for information delivery. Therefore, from an epistemological point of view, there is some room for research on contextual computing in tourism.

## 3. Contextual Computing in Tourism

**Figure 1.**
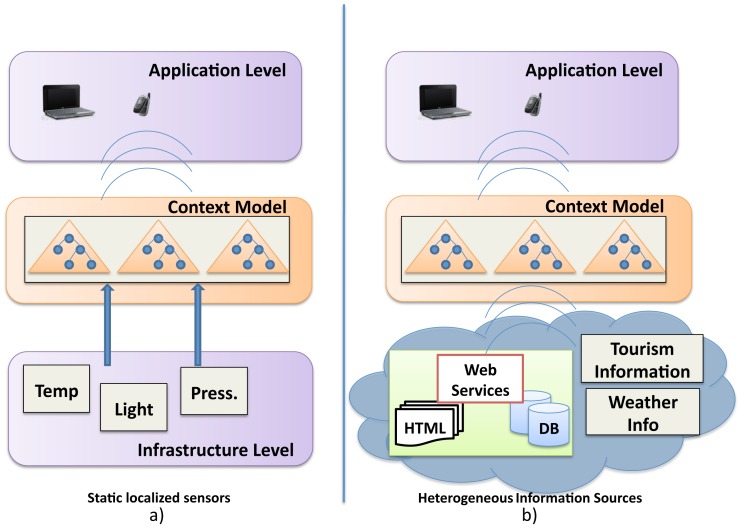
The CONCERT double level interoperability schema.

Two fundamental issues are addressed in order to accomplish the objectives pursued by the CONCERT Framework: Firstly, the conceptual approach to the notion of context in CONCERT is different from the existing ones. CONCERT'S objective is to study the context of visitors in the framework of human mobility in order to more precisely determine the information that formally describes that context, *i.e.*, to establish the requirements in terms of context information. So, the first thing to do is to find out what defines the context of travellers and what minimum information would be needed in order to define that persons context. Then, the following step is to determine where will the source information be retrieved from and how they can be accessed. Secondly, CONCERT proposes a double level interoperability schema (Figure 1).

The first level of interoperability is provided at the infrastructure level and is achieved by not using external sensors to gather contextual information. In order to tackle this barrier, the CONCERT framework gathers part of contextual and tourism data from the Internet as well as from mobile embedded sensors, such as the GPS sensor, thus avoiding some of the major constraints of current context-aware systems. Therefore, CONCERT does not require further complex infrastructures for context information gathering. In this way, having to populate a particular area of interest with sensors would be avoided on the one hand, and on the other, the use of a CONCERT framework based application would not be limited to such areas. The objective is not to contextualize a particular environment but to increase the level of abstraction of context, thus increasing the level of portability and the interoperability of the model by providing the model with the means to gather contextual information anytime, anywhere. In addition, it supports an architecture that is not dependent on the context gathering infrastructure. Moreover, the increasing availability of Open Linked Data populated by sensors deployed in cities and public spaces has to be taken into account.

The second level of interoperability is provided at the model level. It follows similar approaches to other existing ones, *i.e.*, using semantic technologies [10,21–24]. Once the notion of context has been established, it has to be formulated in a consistent computing model in order to effectively process context information. Different context models have been identified and analyzed according to the requirements of ubiquitous computing environments [10,25,26] and context-aware applications [11,27]. Both analyses indicate that ontologies clearly fulfil all requirements and are adequate in order to model contextual information. Besides, ontologies have proved to be good intermediation tools in information integration [28]. In addition, ontologies can also provide reasoning functionalities that are valid for the context model to infer implicit context knowledge. This is crucial to achieve the vision of context pursued by the CONCERT framework. Additionally, in an attempt to increase the level of scalability, modularity and interoperability at the model level, and in order to more accurately meet the requirements of context derived from its conception, context will be modelled in CONCERT by means of a network of ontologies [29].

### 3.1. Definition of Context and Contextual Computing in Tourism

A sound definition of the notion of context is needed in the realm of human mobility. The definition has to clearly delimit the scope of the context framework presented in this paper, and most importantly, to overcome the drawbacks of current conceptions. The previous notions of context consider only particular environments, the ones populated with networks of sensors, which are modelled including the application that runs in them. Thus, existing approaches are too restrictive and make scalability of the concept to other domain applications very cumbersome.

The conception of context presented in this paper suggests to work on context as a main entity and integrated within the framework of human mobility. In addition, it seems that focusing on individuals rather than on systems and their corresponding functionalities can increase the level of abstraction of the system, and as a consequence, it makes the framework more general and potentially more scalable.

The proposed definition adapts and incorporates already existing definitions and makes them more operative for the tourism domain. Thus, based upon Dey's definition [8], context in the framework of human mobility is defined as: *Any relevant information that characterizes the situation of a visitor. A visitor is a traveller taking a trip outside her usual environment and her situation is specified by data concerning (a) the individual itself (b) the individual's environment and surroundings and (c) the individual's objective at a particular moment of time. This information can be of use for a computing-application in order to support the visitor's mobility*.

Accordingly, contextual-computing in tourism *is the scientific approach that studies and observes the context of an individual on the move and pursues to generate knowledge out of that observation in terms of how to model an individual's Context and how to manage information originated in that Context. It also explores how that information can be processed in a way that is useful to assist the visitor. Furthermore, it provides the foundational means to study the way visitors will interact in complex digital environments*.

### 3.2. Context Modelling Ontology in Tourism: ContOlogy

The context ontology ContOlogy is the core element of this framework. It represents the translation of the conceptual notion of context presented earlier into a computing model through an ontology language capable of checking the model's consistency and providing relevant information through rule-based reasoning. Existing context ontologies have been developed for various uses and cover different domains, but they are not general enough and their extensibility and re-use within other frameworks, including human mobility, poses serious difficulties.

**Figure 2.**
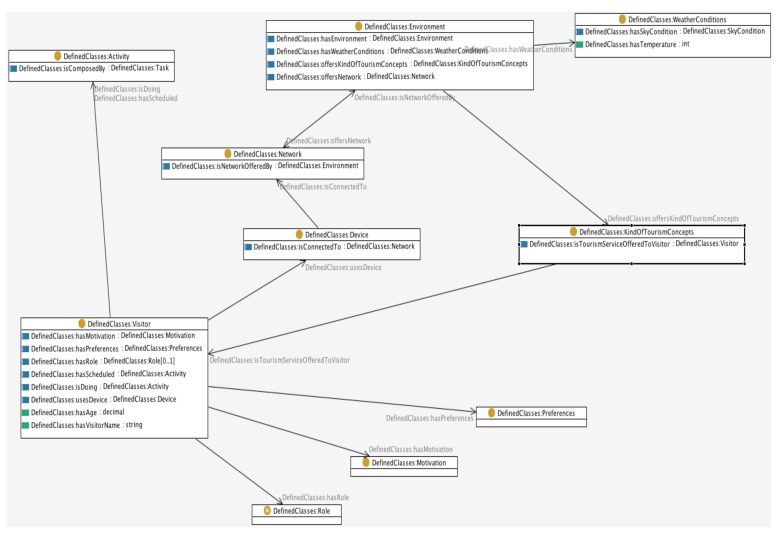
High level view of the ContOlogy network of ontologies.

Following the promises of Ubiquitous Computing, the objective of CONCERT is to assist visitors anytime, anywhere. Since existing ontological resources do not fully fulfil this framework's requirements, a new ontological structure needs to be found. A possible approach to overcome this obstacle is to have a new ontological definition based on networks of ontologies [29]. Thus, the network of ontologies focuses on the different constituents of context (see Figure 2, which shows a high level overview of the ContOlogy network of ontologies) according to the definition provided earlier and it develops or re-uses existing ontologies for each of them, thus adapting to the modular definition of context. Moreover, networks of ontologies enhance ontologies' modularity and flexibility and hence make their interoperability and re-use much simpler and less dependent on the specific purpose of the ontology.

The components of the network of ontologies, *i.e.*, context constituents have been determined considering the definition of the notion of context put forward earlier in the paper, its architecture (see next section) and well established tourism scientific recommendations [7]. Each of these constituents will determine each of the ontologies of the network. The relationship amongst them have been determined based upon the objective pursued by the prototype.

Altogether there are 86 classes, 41 object properties, 22 datatype properties, and 43 restrictions. Figure 2 shows the most important entities that configure the network. The language used to specify each of the ontologies has been OWL in its DL sublanguage [30]. The level of expressivity shown by the network of ontology is SHOIN(D).

## 4. Concert Architecture

This section presents the technical details of the CONCERT Framework, including its architecture, workflow and the rule-based information filtering process.

### 4.1. Framework Architecture

**Figure 3.**
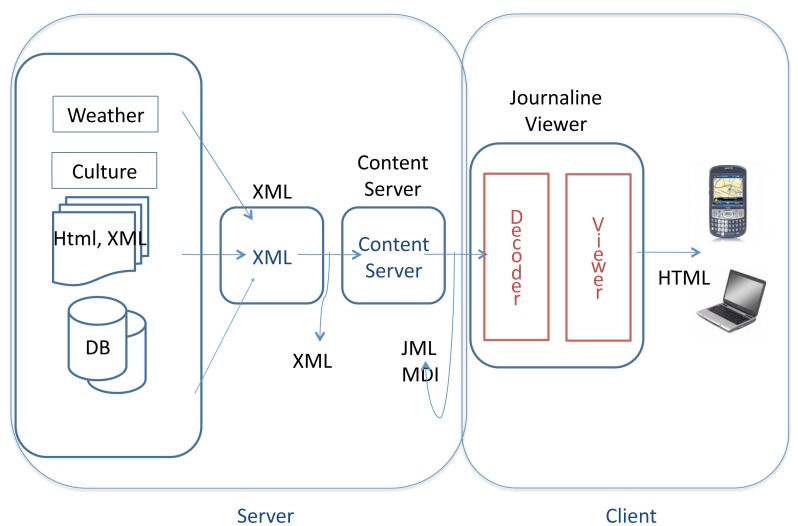
Architecture of Digital Broadcasting.

The conception of the contextual computing framework presented in this paper has two important architectural implications. Firstly, the server side of the CONCERT Framework retrieves contextual and tourism information from the Internet. Secondly, this information is then sent to mobile devices (client side) through digital broadcasting, *i.e.*, there is no uplink communication channel between the client and server sides. This is depicted in Figure 3, following a typical digital broadcasting architecture. Therefore, the contextual computing architecture module needs to necessarily be hosted in the client, where all the reasoning will occur and where all the personal information will be stored.

**Figure 4.**
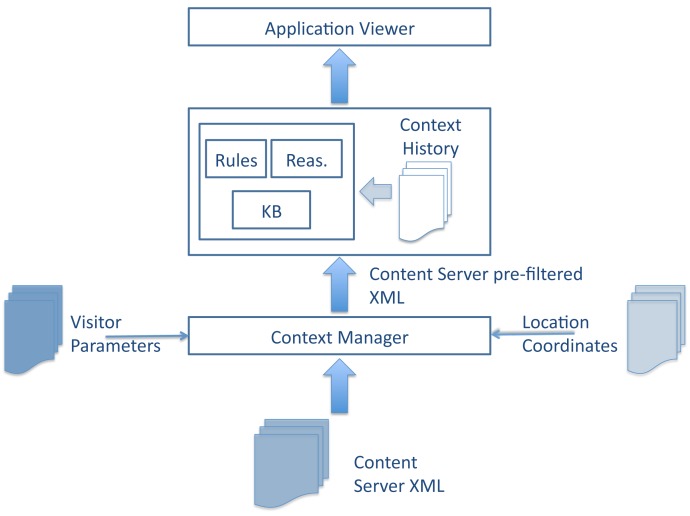
Architecture of Contextual Computing in Tourism.

However, this way of sending tourism information would not be of much help to visitors, since it has not been processed taking into account their specific situational context. Thus, the contextual computing module (Figure 4) extends the previous architecture by acting directly upon the information that is received in the client side and processing it according to the specific situation a visitor may be under.

**Figure 5.**
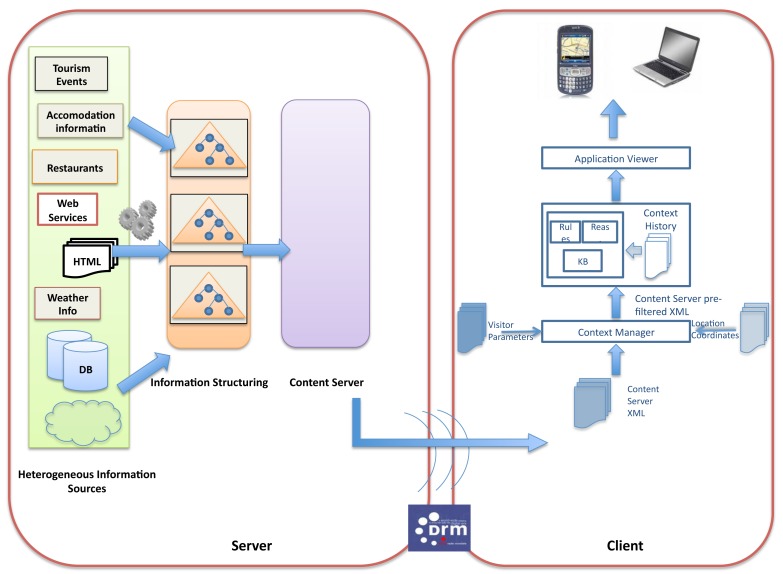
CONCERT Architecture.

The Context Manager is the central component of the Contextual Computing layer. This software module is a background process that runs on mobile devices (client), see Figure 5. The Context Manager receives information from the Context Providers (information input), such as the location sensor embedded in the mobile device, the wireless broadcast source and the user preferences, provided manually by the user. The Context Manager pre-processes all of the gathered information by the location provided by the GPS sensor embedded in the mobile device and consequently feeds the Knowledge Base with that information triggering the flow of events, which are necessary to provide visitors with relevant information for their needs. It offers a centralised way to access context data sources. In addition to this, the Context Manager also runs another service, which consists of removing all of the incoming information at regular intervals in order to avoid information overloads in the knowledge base due to the existence of too many instances.

The knowledge base (KB), *i.e.*, the network of ontologies, is populated with information regarding both visitor parameters and tourism services offered in that environment. There are two other components that interact with the knowledge base, namely the inference rules and a reasoning engine. The latter processes the information by means of the rules and produces a final XML file that contains the context-based information.

**Figure 6.**
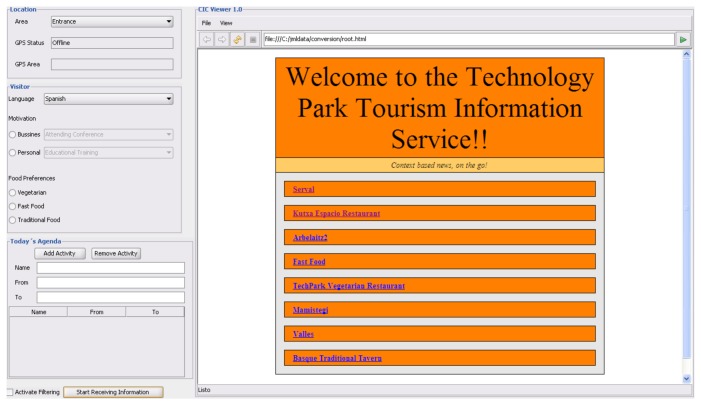
Display of the application

Finally, the application layer consists of the presentation logic that interfaces between the contextual-computing system and the user (see Figure 6). The application features an embedded web browser and handles the input of user information concerning user preferences, content filtering and navigation of the tourism information produced by the knowledge base.

After the incoming information has been filtered by the contextual computing module, it is then ready to be displayed in the visitor's mobile device. The resulting architecture, *i.e.*, the CONCERT Framework Architecture, is depicted in Figure 5.

### 4.2. System Workflow and Filtering Process

One of the objectives of the proposed framework is to filter the tourism information that mobile devices receive. To realize this goal, CONCERT uses a set of semantic-based rules by means of Jena2 Inference Support API. This is a generic purpose rule-based reasoner, used to implement RDFS and OWL reasoners as well. This reasoner supports inference over RDF Graphs and provides forward chaining, backward chaining and a hybrid execution model.

Following, the whole semantic rule-based generic reasoning process (*i.e.*, filtering) is explained step by step as suggested by [31]:

**Step 1:** The visitor turns the CONCERT Framework-based application on. The application automatically detects the visitor's location. In addition to that, the visitor has to manually introduce his/her personal information. This information includes preferred language, motivation of the journey and food preferences. The application can also automatically read the visitor's agenda to consider potential time constraints. Should the agenda information not be reachable or be non-existent, then the visitor could introduce this information manually as well. The personal information dialogue has been designed following the structure the received information has to follow. This way, according to the Contextual computing architecture two of the three incoming parameters to the context manager (*i.e.*, location and personal preferences) are provided to the system.
**Step 2:** The visitor then receives the information that is available in the tourism information system. This information is received in the mobile device through the digital broadcasting Digital Radio Mondiale (DRM) standard, in the DRM dissemination protocol Multimedia Distribution Interface (MDI) following a particular XML-based structure based on keywords, called Journaline Markup Language (JML) [32]. The JML is compressed version of XML information especially structured in order to be sent through MDI. This JML file contains not only general tourism information, but also additional context information to that provided manually by the visitor, such as weather conditions at that particular location, collected from the Internet.
**Step 3:** The Journaline viewer, in the client side, has to decode the incoming information in JML into XML files. The objective of this step is to provide a format of the information that is easily manipulable by the context manager.
**Step 4:** The Context Manager (Figure 4) filters the information that has received by location and generates a location-based XML file containing tourism information. Following, the context manager checks the status of the knowledge base. If it has some content from previous use, it removes it from the knowledge base and stores it in the context history repository, and then it fills the knowledge base with the new XML file together with the user's personal information. Otherwise, the mapping is done straightforwardly. The mapping process is completed through matching predefined keywords of the XML file as individuals in their corresponding classes within the ontology in order to simplify data traffic between the context manager and the knowledge base.
**Step 5:** The instances that the Context Manager has provided to the ontology are the ones that are used to perform the information filtering process and to generate a context-based tourism information XML file. Once the situational context has been loaded on the knowledge base, the rule-based reasoning process begins by means of the rules, an example of which can be seen below.
[ServiceOfferedToVisitor: (?v rdf:type del:Visitor)
(?v del:usesDevice ?d)
… other rules …
(?e del:offersTourismConcepts ?s)
⇒
print(?s, del:isServiceOffered, ?v)]
  Each of the variables is depicted with a question mark. The part of the rule before the arrow is the antecedent and the one after is the consequent. If and only if the entire antecedent is true, the consequent is triggered as an action, *i.e.*, as potential information to be displayed in the screen of the visitor's mobile device.
  The premise of this way of calculating the triggering events is that all the preferences are given the same weights. The mathematical method is described as follows. Let be *n* criteria ${\left\{c_{k}\right\}}_{k=1}^{n}$ and a set *A* = {*a*_1_,_…_, *a_m_*} of *m* alternatives or options, *i.e.*, the tourism services offered in a particular environment. Let be the function *U_k_: A* → {0, 1} such that *U_k_*(*a_i_*) = 1 if *a_i_* satisfies condition *k* and *U_k_*(*a_i_*) = 0, otherwise, for each criteria *k* = 1,…, *n*.
  If all the criteria are equally important, the basic utility function is defined as
  $$\begin{array}{cc} V:A\to & \left\{0,\ldots ,n\right\}\\ a_{i}\to & \sum_{k}U_{k}(a_{i}) \end{array}$$
  and chosen alternatives are those whose utility function is maximal, *i.e., V*(*a_i_*) = max.*_j_ V*(*a_j_*). In the case that preferences and conditions of execution of the triggered service are ordered by their importance, *c*_1_ *> c*_2_ > *c*_3_ > ⋯> *c_n_*, the lexicographic method (see [33–35] among many others) to run the rules is applied. A reciprocal preference relation *R* is defined on the set of alternatives *A* as follows
  $$\begin{array}{cc} R:A\times A\to & \left\{0,0.5,1\right\}\\ \left(a_{i},a_{j}\right)\to & R(a_{i},a_{j})=R_{ij} \end{array}$$
  where
  $$\{\begin{array}{ll} R_{ij}=1 & \text{if}\;U_{k}(a_{i})\geq U_{k}(a_{i})\;\text{for all}\;k\leq h-1\;\text{and}\;U_{h}(a_{i})>U_{h}(a_{j})\;\text{for some criteria}\;h\\ R_{ij}=0.5 & \text{if for all criteria}\;k,U_{k}(a_{i})=U_{k}(a_{j}).\\ R_{ij}=0 & \text{otherwise}. \end{array}$$

Let us recall that reciprocal means that *R_ij_* + *R_ij_* = 1. Therefore, it suffices to observe *R_ij_* to get *R_ji_*. From this relation, the set *G*(*A, R*) of *R*-greatest elements can be considered (see [36]), *G*(*A, R*) = {*a_i_* ∈ *A* | *R_ij_* ≥ 0.5 for all *j* ∈ [1, *n*]}.

**Step 6:** The XML file is further transformed into HTML and it would then be ready to be displayed in any Web-browser.

## 5. Evaluation

This section shows the results of the experiments carried out at CICtourGUNE's lab to validate this research work. The first step is testing the system, *i.e.*, finding out how the system works with respect to its mechanics. This allows to know whether semantic technologies can be used to filter digital broadcast information. This is presented in the technical evaluation. Next is to find out how useful this is in reality for visitors, *i.e.*, whether the mechanics actually produce results that are of any use for tourists. This is presented in the user evaluation.

### 5.1. Technical and Performance Evaluation

The technical evaluation has been carried out according to various critical variables, such as number of rules, processing time, compilation time, heap memory and CPU usage. The importance of these kinds of evaluations is not merely to analyse current performance, but to be able to predict the system's performance in future potential scenarios as well [37]. The experiments have been conducted on a Hewlett Packard laptop. This machine runs on a Microsoft Windows XP Operating System, Version 2002 SP 3 on Intel Core 2 Duo at 1.86 GHz with 4 GB of RAM memory.

The performance experiments have been carried out populating the network of ontologies with a more or less constant number of instances (73 in this case) and running each time more number of and more complex rules on it. The Context Manager constantly checks the existence of instances in the classes of the ontology before introducing a new one, thus minimizing the number of simultaneously existing instances in the ontology and the computational load it would entail [22]. Consequently, the number of instances has not been considered critical and the evaluation has rather focused on the number of rules executed in each of the experiments, while maintaining the order of magnitude of the triples. The reasoning tasks preformed in the ontology correspond to checking whether a number of statements are true in order to execute a certain command.

The average processing time is relatively high, 7.4 s. However, for non-time-critical applications it is under reasonable margins. Nonetheless, the amount of memory consumed (42.37 Mb) is high as expected. There is a lot of room for improvement in that regard, not only in having to reduce the amount of memory consumption in the reasoning process, but also, in making a more efficient use of the CPU, which is rather low (13.72%). Semantic technologies are very beneficial in terms of what can be achieved with them. On the contrary, their computational requirements are extremely high.

### 5.2. User Evaluation

User technology acceptance has become an increasingly studied topic in information systems [38-40]. Much of the previous research in this realm has used the technology acceptance model, TAM [41]. Due to the importance of concepts such as Perceived Utility (PU) [41] and Perceived Ease Of Use (PEOU) [41], from a methodological perspective, the survey used for this user evaluation was designed on the TAM literature, and in particular, it was adapted from David's studies [41,42]. The measurement for behavioural intention and background level were adapted from another experiment work performed by [38] and [43].

An experiment has been conducted on the functionality of the CONCERT framework-based application. The objective of this experiment was to test people's reaction to it and to automatically obtaining context-dependant information. In the following, the conditions in which the experiment was performed is explained.

Altogether 30 participants freely volunteered to take part in the experiment. 56% of them were males. 40% were younger than 36. They all had as different backgrounds and competencies as possible. It is important to bear in mind that the CONCERT framework aims at being used by anyone, regardless of how skilful one is with technology. Therefore, the large variance on experience with technology accounts for a very diverse sample that enhances the final results. The educational level of the sample is relatively high, since 56% of the participants had some kind of university degree. The experiment was carried out with a PC laptop, the same used for the technical evaluation.

The simulation was carried out with a PC laptop. It is the same one in which one set of the observations corresponding to the technical evaluation was performed. The laptop used runs on a Microsoft Windows XP Operating System, Version 2002 SP 3 on Intel Core 2 Duo at 1.86 GHz with 4 GB of RAM memory.

Tourism information was created following the Digital Broadcasting standards and uploaded to the Content Server emulator. The laboratory Content Server broadcasted this information to a particular IP address within the local network area at the lab where experiments were carried out. Therefore, the IP of the laptop had been provided to the Content Server emulator and thus, the laptop could be continuously receiving tourism and context information.

A survey was developed in order to evaluate the performance of the system, in this case, from the perspective of pretending tourists. In addition, participants were given a questionnaire they had to fill in once they had finished their experience with the CONCERT framework-based application.

User testing was conducted in a controlled laboratory environment. First of all, the users were briefly introduced to the CONCERT framework, to the application and to the experiment's objectives. They were instructed on how to interact with the CONCERT framework-based application and before starting with the experience, they were given an example on how to manipulate the application. In addition to that, participants were given a number of scenarios, in which they had to be virtually immersed and try the different options of the application. After having finished the instructions, participants were left on their own in order to accomplish the experience.

Participants were asked to put themselves into the position of a tourist and were also encouraged to recreate, as much as they possibly could, the scenarios that were given. Users had to introduce different types of personal information into the application. By changing their personal context's variables and introducing new ones, they had to experiment with the result the application would provide them with after the rule-based processing of the information by the ontology.

The experiment consisted of seven scenarios and the users had to experiment on all 7 of them. Each of them recreated different contexts or situations and therefore ought to provide different tourism recommendations.

After having completed the user experience, each user had to fill out the above-mentioned questionnaire. On average, the whole experiment took about 15 minutes to be completed.

Results show that 56% of the participants strongly agrees that the CONCERT framework-based application supported them on the move, whereas 44% agrees on that same matter. 68% of the participants expressed that the CONCERT framework-based application improved their tourism experience, whereas only 4% argued that it had no impact on it whatsoever. The application allowed to 60% of the participants to more efficiently move around and 76% of the participants said that the application made it easier for them to find what they needed.

Regarding the ease of use of the application, *i.e.*, the PEOU construct, 40% strong agrees that it is easy to provide personal information to the CONCERT framework based application, 36% agrees and 20% disagrees.

## 6. Conclusions

Current state-of-the-art of mobile devices and communication technologies allow visitors to be connected to sources of information in an anytime/anywhere manner. However, finding useful information as a tourist in an unfamiliar place is not easy due to the lack of familiarity with the location on the one hand and to the interaction restrictions with mobile devices on the other. This paper presents a specific context-aware approach that comprehends the particular characteristics and requirements of context information management in tourism and thus contextualizes the visitor and the visitor's situation.

The experiments carried out have focused on elucidating whether digital broadcasting is (or not) an appropriate technology to disseminate tourism information and then if it can be processed by means of semantic-based rules. Evidence obtained from the experiments has corroborated this issue. However, due to the high memory requirements and low performance of the CPU, further research is needed in order to reduce the size of the ontology, and therefore, reduce the requirements in terms of memory and resource consumptions to perform the reasoning. Additionally, apart from the low data transfer rate, typical mobile phones are not yet equipped with digital broadcast decoders.

The proposed framework observes the nature of human mobility and opens new chances to study complex scenarios in highly digitalized and hybrid spaces. As Internet-based context information sources is a growing reality, there will be no restrictions with respect to the areas where the framework can be used. Other future research will concern the application of fuzzy logic algorithms to the reasoning process. The impact of the Internet of Things in the model of context and in the context and tourism information gathering is also something to be explored. One final point in the agenda for future research is Cloud Computing and the fast emerging field of mobile apps.
